# Latent class trajectories of socioeconomic position over four time points and mortality: the Uppsala Birth Cohort Study

**DOI:** 10.1093/eurpub/ckac060

**Published:** 2022-07-05

**Authors:** Muhammad Zakir Hossin, Amy Heshmati, Ilona Koupil, Anna Goodman, Gita D Mishra

**Affiliations:** Clinical Epidemiology Division, Department of Medicine (Solna), Karolinska Institute, Stockholm, Sweden; Department of Global Public Health, Karolinska Institute, Stockholm, Sweden; Department of Global Public Health, Karolinska Institute, Stockholm, Sweden; Department of Public Health Sciences, Stockholm University, Stockholm, Sweden; Department of Global Public Health, Karolinska Institute, Stockholm, Sweden; Department of Public Health Sciences, Stockholm University, Stockholm, Sweden; Department of Public Health Sciences, Stockholm University, Stockholm, Sweden; Department of Epidemiology and Population Health, London School of Hygiene and Tropical Medicine, London, UK; School of Public Health, The University of Queensland, Herston, Australia

## Abstract

**Background:**

The study assessed socioeconomic position (SEP) over four time points and employed a latent class analysis (LCA) to explore the associations between longitudinal SEP trajectories and late-life mortality.

**Methods:**

We analyzed a cohort of 11 336 members born at the Uppsala University Hospital, Sweden during 1915–29 and followed up for mortality during 1980–2008. SEP was measured at birth, age 10, mid-adulthood and late adulthood. LCA was used to identify SEP trajectories, which were linked to all-cause and cause-specific mortality through Cox proportional hazard regression models.

**Results:**

The age and birth cohort adjusted hazard ratio (HR) of all-cause mortality among the upwardly mobile from middle vs. stable low SEP was 28% lower in men [HR: 0.72; 95% confidence interval (95% CI): 0.65, 0.81] and 30% lower in women (HR: 0.70; 95% CI: 0.62, 0.78). The corresponding HR of cardiovascular mortality was 30% lower in men (HR: 0.70; 95% CI: 0.60, 0.82) and 31% lower in women (HR: 0.69; 95% CI: 0.58, 0.83). Upward mobility was also associated with decreased HR of mortality from respiratory diseases and injuries among men and from cancer, respiratory diseases, injuries and mental disorders among women. The upwardly mobile were similar to the stable high group in terms of their HRs of mortality from all-causes and cardiovascular, cancer and mental diseases.

**Conclusions:**

Upward mobility appeared to be protective of mortality from a wide range of causes. Interventions aiming to prevent deaths can benefit from creating optimal conditions earlier in the life course, letting disadvantaged children maximize their socioeconomic and health potentials.

## Introduction

Socioeconomic position (SEP) is associated with morbidity and mortality across all stages of the life course, whereby those from disadvantaged backgrounds have poorer health outcomes.^1–5^ Acknowledging the importance of one’s SEP throughout the life course enables us to ascertain when the exposure has the most effect and where interventions can be focused.

Social mobility, i.e. movement from one social class to another, can also affect an individual’s health. Many studies have investigated the relationship between social mobility and mortality in later life in Sweden.^6–14^ In most of these studies, individuals who were downwardly mobile had higher mortality than those who were always advantaged, but not necessarily more so than individuals who were always disadvantaged,^7^^,^^9^^,^^11–13^ suggesting that cumulative class experience can have a more profound impact on health than childhood or adulthood SEP.^15^ Moreover, some studies have observed that being upwardly mobile leads to higher mortality^8^^,^^9^^,^^11^ while other studies have shown that upward mobility is protective against mortality.^14^^,^^16^ Thus, being socially mobile, regardless of direction, can affect an individual’s health.

Although there have been many studies investigating the effect of social mobility on mortality in Sweden, most studies measured SEP at only two time points^6^^,^^7^^,^^10–14^ and have employed a more traditional methodological approach. One recent study, based on the same cohort as used in this study, measured SEP at four time points with an aim to identify what time period is most important for the effect of SEP on mortality.^17^ However, none of the previous studies have performed latent class analyses when investigating the relationship between SEP and mortality across the life course. The latent class procedure employs a ‘person-centered’ modeling strategy, which might be particularly useful for the identification of distinct trajectories of individuals sharing a common socioeconomic profile over time.^18^^,^^19^

Using data from the first generation of the Uppsala Birth Cohort Multigenerational Study (UBCoS Multigen),^20^ we aimed to explore the relationship between latent class trajectories of SEP and mortality. Specifically, we investigated the effect the temporal trajectories have on (i) all-cause mortality and (ii) cause-specific mortality i.e. mortality from cardiovascular diseases (CVD), cancer, injuries and poisoning, respiratory disease, mental disease and other causes. SEP was measured over four time points—at birth, during childhood (around age 10), in adulthood (ages 30–45) and in later life (ages 50–65).

## Methods

### Study population

The members of the original cohort of UBCoS Multigen, born during 1915–29 in the Uppsala University Hospital in Sweden,^20^ constitute the sample for this study. The cohort consists of 14 192 live born individuals (female 48%), and is considered to be representative of the national infant population born during that period.^21^ Through parish records, it was possible to trace 97% of the cohort (*n* = 13 811) until routine registers became available in the 1960s. The final analytical sample were 11 336 cohort members who were still alive and living in Sweden on 15 September 1980, and who had at least one measure of SEP recorded (5729 males and 5607 females). Ethical approval for the study was obtained from the Regional Ethics Committee in Stockholm.

### Measures

We measured SEP at four time points over the life course: at birth, during childhood (at age 10, ± 5 years), in mid-adulthood (ages 30–45) and in late adulthood (ages 50–65). SEP at birth and at age 10 was primarily based on father’s occupation. Mother’s occupation was used if father’s occupation was not available. The main source of information for SEP at birth was the obstetric records of the child. If this SEP information was missing, we assigned family SEP within 5 years of the child’s birth from sibling’s obstetric records or the Census 1930. Data on SEP at age 10 mostly comes from archived school records during the child’s third year of primary school. Again, if this information was missing, we assigned family SEP within 5 years of age 10 based on obstetric or school records of a sibling or the Census 1930. The Census 1960 provided data on SEP at ages 30–45, which was based on the occupation of the head of the household. Data on SEP at ages 50–65 were derived from Census 1980 and was based on the highest occupation of either the individual or the cohabiting partner.

The Swedish socioeconomic classification of occupation^22^ was used to code SEP at all ages. To create comparable SEP groups across the life course, occupational categories were divided into ‘high’ (high and intermediate non-manual occupations), ‘middle’ (lower non-manual occupations, skilled manual workers, entrepreneurs and farmers) and ‘low’ (unskilled manual workers and those who were non-employed or working part-time, students, housewives or had non-classifiable occupations). The latest SEP was coded as ‘missing’ for those who were already retired and were aged 62 or over, given that it was relatively common (>30%) for the study subjects to have retired from age 62 onwards and the category of retirees was quite heterogeneous. However, we coded SEP as ‘low’ for those who were not working and were under 62 years of age.

Data on all-cause and cause-specific mortality were obtained from the Swedish Causes of Death Registry.^23^ We used the 8th, 9th and 10th revisions of the World Health Organization’s International Classification of Disease (ICD) codes to define cause-specific mortality (Supplementary table S1). All the causes of death were aggregated into six major groups of mortality: CVD, cancer, injury and poisoning, respiratory diseases, mental disorders and Alzheimer’s disease and other causes of mortality. Supplementary table S1 presents the ICD codes for Revisions 8–10 for major diagnoses within each domain of mortality.

We also obtained the dates of emigration from the Total Population database^24^ to censor the study subjects who emigrated during the follow-up period from 1980 to 2008. The obstetric records provided data on gender (men vs. women) and birth years (divided into three groups: 1915–19, 1920–24 and 1925–29), which were used as control variables in the study.

### Statistical analyses

To identify subgroups that have similar patterns of change in SEP over time, we performed a latent class analysis (LCA). The LCA Stata Plugin^25^ was used to assign individuals to their most likely classes given their responses in the observed SEP indicators. This newly developed plugin provides the Stata users with a simplistic approach to estimating latent classes measured by categorical indicators, and has features and functionality identical to the SAS PROC LCA.^18^

In selecting the best latent class model, we fitted a series of LCA models starting with a two-class model and ending with a six-class model. Assuming that the data were missing at random, the LCA procedure used the expectation–maximization algorithm to allow us to include all subjects with at least one measure of SEP and adjusted for the missing data using maximum likelihood estimations.^25^ We selected the five-class solution for both men and women based on the following criteria: Akaike’s information criterion, Bayesian information criterion (BIC), the sample-size-adjusted BIC, the sizes of the latent classes and the substantive meaningfulness of the model (see Supplementary tables S2 and S3). Class sizes were calculated based on the estimated posterior class membership and classes were labeled to summarize the life course SEP trajectories of the respective classes within each gender.

We fitted Cox proportional hazard models with age at the time scale to estimate hazard ratios (HRs) and 95% confidence interval (95% CI) for the associations of selected SEP trajectories with all-cause and cause-specific mortality. Follow-up for mortality began on 15 September 1980 (the date of the 1980 Census from which the latest SEP was derived) and continued until the date of death, emigration or until 31 December 2008. All analyses were stratified by gender and were adjusted for birth year. Additionally, to examine the potential role of cognitive ability in the associations between upward socioeconomic trajectories and mortality outcomes, we adjusted for school grades (measured at age 10 as the mean of standardized scores obtained in 10 individual school subjects)^26^ in a Supplementary analysis (see Supplementary tables S6 and S7).

## Results

Table 1 displays the distribution of SEP at each time point for the sample population. Among both men and women, only a small proportion of the sample belonged to high SEP (<10%) at birth and around age 10. The proportion of high SEP substantially increased in mid-adult life, with a higher increase in women than men (45% vs. 36%). When compared to mid-adulthood, however, the proportion of high SEP declined in later adulthood and the decline was steeper in women than men. The distribution of the missing SEP data according to social class positions in childhood and adulthood is presented in Supplementary table S4. The data suggest that those with high SEP at birth were more likely to have missing data on SEP at age 10 compared to those with low or middle SEP at birth. On the other hand, the proportion of missing data on SEP in late adulthood was slightly higher in those with low SEP at birth or mid-adulthood compared to those with high SEP.

**Table 1:** The distribution (%) of SEP at each time point

Socioeconomic position	Men (*n* = 5729)				Women (*n* = 5607)			
	Birth	Age 10	Age 31–45	Age 51–65	Birth	Age 10	Age 31–45	Age 51–65
Low	50	31	44	22	51	32	30	29
Middle	39	40	16	38	39	38	20	33
High	9	7	36	29	8	7	45	23
Missing	2	22	4	10	2	23	5	15

Supplementary tables S2 and S3 show the latent class fit analysis statistics for SEP trajectories in men and women, respectively. A five-category classification of SEP was selected for both genders: stable high, upwards from middle, stable middle/middle to low, upwards from low and stable low SEP. The selected latent class trajectories in men and women are illustrated in Supplementary figures S1 and S2, respectively. While the proportions of individuals with stable high SEP were similar in both genders, a greater number of women than men tended to be upwardly mobile, as indicated by the proportions in the upwards from middle (18% vs. 13%) and upwards from low (27% vs. 19%) SEP trajectories.

**Figure 1:**
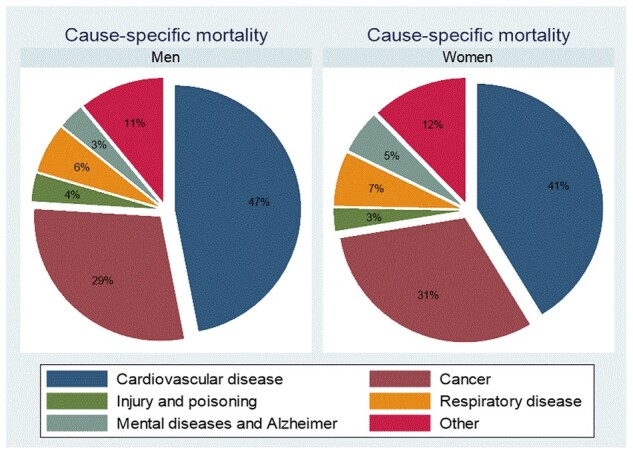
Pie chart showing the distribution of cause-specific mortality outcomes by gender

The study subjects were 50–65 years old at baseline in 1980. During the 28-year follow-up period, a total of 6768 deaths were recorded. The gender-specific distributions of the cause-specific deaths are shown as pie charts in figure 1. Mortality from CVDs dominated the distributions, with cancer being the second leading cause of mortality in both men and women. The major causes of death within each domain of mortality are presented in Supplementary table S5.

Table 2 shows the incidence rates of all-cause and cause-specific mortality by the latent class trajectories. Compared to the stable low SEP trajectory, the stable high SEP trajectory showed lower incidence rates of total mortality and mortality from CVDs in both men and women.

**Table 2:** Incidence rates of mortality by latent class trajectories of SEP in men and women aged 51–95 years

Latent class trajectories of socioeconomic position	Mortality													
	All-cause		Cardiovascular disease		Cancer		Injuries and poisoning		Respiratory disease		Mental disorders		Other	
	No. of death	Rates^a^ (95% CI)	No. of death	Rates^a^ (95% CI)	No. of death	Rates^a^ (95% CI)	No. of death	Rates^a^ (95% CI)	No. of death	Rates^a^ (95% CI)	No. of death	Rates^a^ (95% CI)	No. of death	Rates^a^ (95% CI)
Men (*n* = 5729)														
Stable low	1373	36.6 (34.7, 38.6)	669	17.8 (16.5, 19.3)	352	9.4 (8.5, 10.4)	60	1.6 (1.2, 2.1)	89	2.4 (1.9, 2.9)	42	1.1 (0.8, 1.5)	153	4.1 (3.5, 4.8)
Upward from low	558	29.1 (26.8, 31.6)	277	14.4 (12.8, 16.2)	160	8.3 (7.1, 9.7)	16	0.8 (0.5, 1.4)	30	1.6 (1.1, 2.2)	15	0.8 (0.5, 1.3)	57	3.0 (2.3, 3.9)
Stable middle/middle to low	1126	31.6 (29.8, 33.5)	502	14.1 (12.9, 15.4)	345	9.7 (8.7, 10.8)	37	1.0 (0.8, 1.4)	84	2.4 (1.9, 2.9)	37	1.0 (0.8, 1.4)	117	3.3 (2.7, 3.9)
Upward from middle	449	26.5 (24.1, 29.0)	208	12.3 (10.7, 14.0)	144	8.5 (7.2, 10.0)	9	0.5 (0.3, 1.0)	19	1.1 (0.7, 1.8)	15	0.9 (0.5, 1.5)	53	3.1 (2.4, 4.1)
Stable high	314	27.8 (24.9, 31.1)	130	11.5 (9.7, 13.7)	102	9.0 (7.4, 11.0)	14	1.2 (0.7, 2.1)	20	1.8 (1.1, 2.7)	14	1.2 (0.7, 2.1)	32	2.8 (2.0. 4.0)
Women (*n* = 5607)														
Stable low	831	26.7 (24.9, 28.5)	336	10.8 (9.6, 12.0)	247	7.9 (7.0, 9.0)	27	0.9 (0.6, 1.3)	69	2.2 (1.7, 2.8)	47	1.5 (1.1, 2.0)	105	3.4 (2.8, 4.1)
Upward from low	623	19.1 (17.6, 20.6)	257	7.9 (7.0, 8.9)	210	6.4 (5.6, 7.4)	13	0.4 (0.2, 0.7)	39	1.2 (0.9, 1.6)	32	1.0 (0.7, 1.4)	72	2.2 (1.7, 2.8)
Stable middle/middle to low	801	22.4 (20.9, 24.1)	352	9.9 (8.9, 11.0)	230	6.4 (5.7, 7.3)	26	0.7 (0.5, 1.1)	42	1.2 (0.9, 1.6)	50	1.4 (1.1, 1.8)	100	2.8 (2.3, 3.4)
Upward from middle	451	19.4 (17.7, 21.3)	180	7.7 (6.7, 9.0)	154	6.6 (5.7, 7.8)	16	0.7 (0.4, 1.1)	30	1.3 (0.9, 1.8)	22	0.9 (0.6, 1.4)	49	2.1 (1.6, 2.8)
Stable high	242	20.8 (18.4, 23.6)	94	8.1 (6.6, 9.9)	74	6.4 (5.1, 8.0)	4	0.3 (0.1, 0.9)	26	2.2 (1.5, 3.3)	11	0.9 (0.5, 1.7)	33	2.8 (2.0, 4.0)

Note: Mortality from mental disorders includes Alzheimer’s disease mortality.

a Age-adjusted incidence rates per 1000.

The HRs for all-cause and cause-specific mortality by latent class trajectories of SEP are presented in table 3. The SEP trajectories showed similar patterns of associations with both all-cause and CVD mortality. Compared to the stable low trajectory, the stable high trajectory showed 25% lower HR of all-cause mortality in men (HR 0.75, 95% CI: 0.67, 0.85) and 27% lower HR in women (HR 0.73, 95% CI: 0.63, 0.84). The HR of CVD mortality associated with stable high SEP were 35% lower in men (HR 0.65, 95% CI: 0.53, 0.78) and 30% lower in women (HR 0.70, 95% CI: 0.56, 0.88). Similarly, the upwardly mobile men and women—both upwards from low and upwards from middle—were found to have lower HR of mortality from all-causes and CVDs compared to the stable low SEP trajectory. The HRs in the upward mobility trajectories were almost similar to the HRs in the stable high trajectory, especially among the women. Among men, however, the HR of CVD mortality in the upward from low trajectory decreased by 13% while the HR in the upward from middle trajectory decreased by 30% in relation to the stable low trajectory.

**Table 3:** HRs (95% CI) of the associations between latent class trajectories of SEP and mortality in men and women aged 51–95 years

Latent class trajectories of socioeconomic position	Mortality						
	All-cause	Cardiovascular disease	Cancer	Injuries & poisoning	Respiratory disease	Mental disorders	Other
	HR (95% CI)	HR (95% CI)	HR (95% CI)	HR (95% CI)	HR (95% CI)	HR (95% CI)	HR (95% CI)
Men (*n* = 5729)							
No. of death	3820	1786	1103	136	242	123	412
Stable low (reference)	1.00	1.00	1.00	1.00	1.00	1.00	1.00
Upward from low	0.83 (0.75, 0.92)	0.87 (0.75, 0.99)	090 (0.75, 1.09)	0.53 (0.30, 0.93)	0.71 (0.47, 1.08)	0.71 (0.39, 1.28)	0.76 (0.56, 1.03)
Stable middle/middle to low	0.87 (0.80, 0.94)	0.80 (0.71, 0.90)	1.03 (0.89, 1.19)	0.65 (0.43, 0.98)	1.00 (0.74, 1.35)	0.92 (0.59, 1.43)	0.81 (0.63, 1.03)
Upward from middle	0.72 (0.65, 0.81)	0.70 (0.60, 0.82)	0.89 (0.74, 1.09)	0.33 (0.16, 0.66)	0.48 (0.29, 0.78)	0.76 (0.42, 1.37)	0.76 (0.55, 1.04)
Stable high	0.75 (0.67, 0.85)	0.65 (0.53, 0.78)	0.95 (0.77, 1.19)	0.76 (0.42, 1.36)	0.74 (0.46, 1.21)	1.05 (0.58, 1.93)	0.68 (0.46, 0.99)
*P*-value for heterogeneity*	0.001	0.001	0.51	0.009	0.018	0.722	0.117
Women (*n* = 5607)							
No. of death	2948	1219	915	86	206	162	359
Stable low (reference)	1.00	1.00	1.00	1.00	1.00	1.00	1.00
Upward from low	0.68 (0.61, 0.76)	0.70 (0.59, 0.82)	0.80 (0.66, 0.96)	0.43 (0.22–0.83)	0.50 (0.34, 0.75)	0.57 (0.37, 0.90)	0.61 (0.45, 0.82)
Stable middle/middle to low	0.80 (0.72, 0.88)	0.86 (0.74, 0.99)	0.79 (0.66, 0.95)	0.79 (0.46, 1.35)	0.50 (0.34, 0.73)	0.82 (0.55, 1.22)	0.77 (0.59, 1.02)
Upward from middle	0.70 (0.62, 0.78)	0.69 (0.58, 0.83)	0.82 (0.67, 0.99)	0.75 (0.41, 1.40)	0.55 (0.36, 0.84)	0.57 (0.35, 0.95)	0.59 (0.42, 0.83)
Stable high	0.73 (0.63, 0.84)	0.70 (0.56, 0.88)	0.78 (0.60, 1.02)	0.37 (0.13, 1.05)	0.94 (0.60, 1.47)	0.53 (0.28, 1.03)	0.77 (0.52, 1.33)
*P*-value for heterogeneity*	0.012	0.01	0.056	0.076	0.001	0.052	0.006

Note: Mortality from mental disorders includes Alzheimer’s disease mortality.

HR, hazard ratio; 95% CI, 95% confidence interval.

Analyses are minimally adjusted for age and birth cohort (1915–19, 1920–24, 1925–29).

* *P*-values obtained from Wald test to test the significance of the overall association.

The women with upward from low or middle SEP trajectories had around 20% lower HRs of cancer mortality compared to women with stable low SEP. Regarding mortality from injuries and poisoning, the HRs were substantially smaller among both men (HR 0.53, 95% CI: 0.30, 0.93) and women (HR 0.43, 95% CI: 0.22, 0.83) who were upwardly mobile from low SEP relative to those with stable low SEP. Further, the upward from middle SEP and stable middle/middle to low SEP trajectories were associated with reduced HR of mortality from injuries and poisoning in men (table 3).

The upward from middle SEP vs. stable low SEP showed very low HRs of mortality from respiratory diseases in both men (HR 0.48, 95% CI: 0.29, 0.78) and women (HR 0.55, 95% CI: 0.36, 0.84). As for mortality from mental disorders, the HRs among women were found to be smaller in stable high (HR 0.53, 95% CI: 0.28, 1.03), upward from middle (HR 0.57, 95% CI: 0.35, 0.95) and upward from low (HR 0.57, 95% CI: 0.37, 0.90) SEP trajectories compared to the stable low trajectory. There was also an association between the SEP trajectories and mortality from ‘other’ causes in women (*P* < 0.05), with the stable high trajectory showing 39% decreased HR of mortality (HR 0.61; 95% CI: 0.45, 0.82) relative to the stable low trajectory (table 3).

The school grades at age 10, when additionally adjusted for in the Cox regression models, contributed to little or no attenuations of the associations between socioeconomic trajectories and mortality outcomes (Supplementary tables S6 and S7).

## Discussion

We measured SEP over four time points across the life course and aimed to link the latent class trajectories of SEP to the mortality experience of a historical Swedish cohort during a 28-year follow-up period. We found that an advantaged social class throughout the life course was protective of deaths from all-causes and CVDs among both men and women. Upwardly mobile men and women appeared protected from all-cause mortality and mortality from CVDs, respiratory diseases and injuries. Upward mobility was also associated with lower risks of mortality from cancer and mental disorders in women. The observed risk of dying from CVDs, cancer and mental disorders among the upwardly mobile reached as low as in the group with consistently stable high SEP.

Our LCA analysis of the repeated measures of SEP, showing remarkably high proportions of individuals who experienced upward mobility, reflects the changes in the Swedish occupational structure after the Second World War. The labor market in Sweden after the Second World War was characterized by a decline of unskilled work including farming and the expansion of the service sector, which has likely contributed to the observed upward social mobility in both men and women in our study cohort. The decline of unskilled work was shown to be more pronounced in women than men, leading to their greater upward mobility into the service class positions.^27^

The observed lower effects of life-time high SEP on total mortality and mortality from CVD compared to life-time low SEP in this study are partly consistent with the existing literature^28–32^ documenting evidence of an accumulative effect of SEP across the life course. Among men, e.g. upward mobility from low SEP showed lower risk of CVD mortality than stable low SEP, but the observed risk tended to be elevated relative to upward from middle SEP or stable high SEP—a finding that lends indirect support to the hypothesis of accumulation of socioeconomic disadvantages over the life course. Our study further demonstrates that both upward from middle and upward from low trajectories in women and the upward from middle trajectory in men were protective of total mortality and CVD mortality to similar extent as stable high SEP. These findings stand in contrast to previous studies that showed increased risks of CVD mortality associated with upward mobility relative to stable high SEP.^8^^,^^13^ The findings imply that the negative effect of disadvantaged childhood SEP on later mortality, as demonstrated in previous studies,^1^ can be offset by improving socioeconomic conditions in adult life. A similar conclusion was drawn by Heshmati et al.^17^ who used the same data material and aimed to address which of the three life course models—critical, sensitive and accumulation models^33^—was the best fit for the observed data. The study found that the association between SEP across the life course and all-cause mortality is best described by the sensitive period model whereby an advantaged SEP at the latest stage of life (age 50–65 years) showed the largest protective effect.

Few investigations have explored the relationship between socioeconomic trajectories across the life course and mortality from cancer,^16^^,^^29^ depression,^34^^,^^35^ respiratory diseases^36^^,^^37^ and injuries.^16^ The mortality from cancer and mental disorders among the women of our study, like CVD mortality, displayed a similar pattern of association with the socioeconomic trajectories, with the upwardly mobile women closely resembling the stable high group. However, we found no difference in cancer-related or mental illness-related mortality by socioeconomic trajectories in men. Our finding related to cancer mortality among men is partly in accord with a previous study that found no effect of upward or downward mobility on cancer mortality among a cohort of Scottish men.^29^ In contrast, a recent Swedish study documented a protective effect of upward mobility on cancer mortality in men but not in women.^16^ The inverse associations between upward mobility and mortality from respiratory and mental illnesses in this study are comparable with the limited available evidence showing better lung function^36^^,^^37^ and psychological health^34^^,^^35^ among the upwardly mobile individuals. As for mortality due to injuries and poisoning, our study documents for the first time, to our best knowledge, a decreased risk associated with upward mobility.

Individuals belonging to different socioeconomic trajectories can be differentially exposed to the burden of health-risk factors,^38^^,^^39^ which may accumulate over the life course and create mortality divides along the lines of socioeconomic trajectories.^32^ While social class at different stages of the life course might have differential effects on mortality,^15^^,^^17^ the causal processes through which a shift in social class sets the stage for later onset of diseases and mortality inequalities are less clear. A movement from low to high social class position may generally be rewarded by a set of material and social resources, enabling the upwardly mobile individuals to avoid health risks by having better controls over their lives and adopting health promoting behaviors.^40^^,^^41^

On the other hand, Sorokin’s so-called dissociative theory^42^ postulates that the transition from lower to higher class positions may become a stressful experience. Sorokin^42^ argues that the movement to higher- or lower-class positions may mean a weakened tie with the class of origin and a lack of adaptation to the new class of destination, which may lead to social isolation and distress and eventually interfere with the health and wellbeing of the movers. Thus, the dissociative thesis considers social mobility as disruptive and harmful, although the empirical literature has generated rather mixed evidence.^34^^,^^43–45^ Our ability to directly quantify the specific mechanisms in this study is limited. Overall, our study suggests that if there were any negative health consequences of upward mobility, these would be outweighed by its beneficial effects as evident from the fact that the upwardly mobile groups fared almost as likely as the consistently advantaged group for some mortality outcomes.

Social class mobility may also have a selective effect on health and mortality. A health selection means that individuals with better health and personality profiles are more likely to be upwardly mobile. Viewed from this perspective, the association between social mobility and mortality is likely driven by health or health-related factors earlier in the life course.^7^

### Strengths and limitations

In contrast to studies of life course socioeconomic trajectories that are based on social class measured at two time points—childhood and adulthood—we measured social class at four distinct life stages and used a model-based approach to identify latent groups of individuals who share a common socioeconomic profile throughout the life cycle. Previous research predominantly employed variable-centered strategies to construct socioeconomic trajectories. In contrast, the person-centered strategy of the latent class modeling procedure has given this study a unique advantage to provide finer understanding of individuals’ movement in the socioeconomic hierarchy over time. Furthermore, to our knowledge, this is the most comprehensive study thus far that has been able to systematically link mortality from a wide variety of causes to the longitudinal socioeconomic trajectories separately for men and women. Another strength of the study is the use of register-based data, especially the Swedish Cause of Death Registry, which is an excellent source of high quality and virtually complete data on all deaths in Sweden and is linked to other national registers through unique personal identification numbers.^46^ Consequently, it has enhanced the internal validity of the study by ruling out the possibility of attrition bias that could originate from systematic losses to follow-up.

The LCA approach, although appealing and widely used, is not without its limitations. It identifies subgroups of individuals who are assumed to be homogeneous within their respective groups. Violation of this assumption may lead to poor model performance and misclassification of the latent classes.^47^ As LCA models are often based on repeated measures, incomplete data are quite common and are often handled under the assumption that the data are missing at random.^19^^,^^48^ If missingness depends on unmeasured covariates, the assumption may not hold, potentially resulting in biased model estimates.

A weakness of our study is inability to disentangle the health selection effects from the effects of socioeconomic trajectories. Because childhood health conditions and other common background factors may affect both socioeconomic trajectories and mortality outcomes, the estimated associations may reflect both social causation and health selection. Interestingly, a recent empirical investigation of Swedish data has shown that prior health plays a minor role in the association between social mobility and mortality.^49^ Further, while the upwardly mobile individuals may carry forward some of the disadvantages inherited from the class of origin, we could not isolate the class effects from the effects of upward mobility itself.

## Conclusions

Using a unique methodological strategy, our study demonstrates strong evidence of associations between latent class trajectories of SEP and mortality outcomes, with the stable high and upward socioeconomic trajectories showing reduced risks of all-cause and CVD mortality in both men and women compared to the stable low trajectory. A striking finding was that upward mobility is protective of mortality from CVDs, cancer, respiratory diseases and mental illness among women and from CVDs, injuries and poisoning and respiratory diseases among men. Public health strategies aiming to promote social mobility and prevent avoidable deaths should improve living conditions and life chances from early in the life course by targeting socioeconomically disadvantaged families.

## Author contributions

M.Z.H. took the lead in drafting the manuscript. I.K. and G.D.M. conceived the study; A.G., A.H. and M.Z.H. took part in further development of the study design. A.G. and I.K. processed the data for analysis. G.D.M., A.G. and A.H. carried out the preliminary analyses. M.Z.H. performed the final statistical analysis with advice from G.D.M. and I.K. All authors contributed to writing, critical reading and revisions of the manuscript.

## Supplementary data

Supplementary data are available at *EURPUB* online.

## Funding

The research leading to these results received funding from the Swedish Research Council for Health, Working Life and Welfare under Grant Agreement No. 2018-00211 and from the European Union’s Horizon 2020 research and innovation programme under Grant Agreement No. 635 316 (ATHLOS project).

*Conflicts of interest*: None declared.

Key points

- Most studies that investigated the association between life course socioeconomic position and mortality lacked data on socioeconomic position over multiple timepoints and used traditional methodological approaches.
- This study assessed socioeconomic position at four distinct life stages and used a latent class approach to identify subgroups of individuals sharing a common socioeconomic profile over the life course.
- Upward mobility appears to be protective of all-cause mortality and mortality from a range of causes.
- The upwardly mobile were close to the stable high socioeconomic group in terms of the level of protection against certain mortality outcomes.
- Public health strategies should promote social mobility by improving living conditions and life chances of children born in socioeconomically disadvantaged families.

## Supplementary Material

ckac060_Supplementary_Data

## Notes

References [41–49] are provided in the Supplementary data.
