# Supplementary material for: Latent class trajectories of socioeconomic position over four time points and mortality: the Uppsala Birth Cohort Study
Source: Eur J Public Health. 2022 Jul 5;32(4):522–7. doi: 10.1093/eurpub/ckac060 (PMC9341739; doi:10.1093/eurpub/ckac060)
Supplement: ckac060_Supplementary_Data [file ckac060_supplementary_data.zip › ejph-2021-07-om-0780-File003.docx]

**Supplementary Contents**

**Additional References:** 41 through 49

**Figure S1.** Latent class trajectories of socioeconomic position in men (n=5729)

**Figure S2.** Latent class trajectories of socioeconomic position in women (n=5607)

**Table S1.** International Classification of Disease Codes for broad groups of mortality with major diagnoses in each group

**Table S2.** Goodness-of-fit criteria for different latent class models of socioeconomic trajectories in men (n=5729)

**Table S3.** Goodness-of-fit criteria for different latent class models of socioeconomic trajectories in women (n=5607)

**Table S4.** The distribution (%) of missing cases of socioeconomic position according to socioeconomic position at birth and in adulthood (based on the study sample who survived till the start of follow up in 1980)

**Table S5.** The distribution of major diagnoses within the domains of cause-specific mortality

**Table S6**. Hazard ratios (95% CI) of the associations between latent class trajectories of socioeconomic position and mortality in men aged 51-95 years: contribution of school grades (n=4716)

**Table S7**. Hazard ratios (95% CI) of the associations between latent class trajectories of socioeconomic position and mortality in women aged 51-95 years: contribution of school grades (n=4642

**Additional References:** 41 through 49

41. Gall SL, Abbott-Chapman J, Patton GC, Dwyer T, Venn A. Intergenerational educational mobility is associated with cardiovascular disease risk behaviours in a cohort of young Australian adults: The Childhood Determinants of Adult Health (CDAH) Study. BMC Public Health. 2010;10.

42. Sorokin PA. Social and Cultural Mobility. 4th ed. New York: Free Press; 1959. Pp. 99–145

43. Jonsson F, Sebastian MS, Hammarström A, Gustafsson PE. Intragenerational social mobility and functional somatic symptoms in a northern Swedish context: analyses of diagonal reference models. Int J Equity Health. 2017;16(1):1–10.

44. Chan TW. Social mobility and the well‐being of individuals. Br J Sociol. 2018;69(1):183–206.

45. Ellis RA, Lane WC. Social mobility and social isolation: A test of Sorokin’s dissociative hypothesis. Am Sociol Rev. 1967;32(2):237.

46. Brooke HL, Talbäck M, Hörnblad J, Johansson LA, Ludvigsson JF, Druid H, et al. The Swedish cause of death register. Eur J Epidemiol. 2017;32(9):765–73.

47. Davies CE, Glonek GFV, Giles LC. The impact of covariance misspecification in group-based trajectory models for longitudinal data with non-stationary covariance structure. Stat Methods Med Res. 2017;26(4):1982–91.

48. Enders CK, Bandalos DL. The relative performance of full information maximum likelihood estimation for missing data in structural equation models. Struct Equ Model. 2001;8(3):430–57.

49. Billingsley S. Sick leave absence and the relationship between intra-generational social mobility and mortality: Health selection in Sweden. BMC Public Health. 2020;20(1):1–15.

**Supplementary Figures**

|  |  |
| --- | --- |
|  |  |
|  |  |

**Figure S1.** Latent class trajectories of socioeconomic position in men (n=5729)

|  |  |
| --- | --- |
|  |  |
|  |  |

**Figure S2.** Latent class trajectories of socioeconomic position in women (n=5607)

**Supplementary Tables**

| **Table S1.** International Classification of Disease Codes for broad groups of mortality with major diagnoses in each group | | | |
| --- | --- | --- | --- |
|  | **ICD-8** (1980-1986) | **ICD-9** (1987-1996) | **ICD-10** (1997-2009) |
| **Cardiovascular disease** | 390-458 | 390-459 | I00-I99 |
| Ischaemic heart disease | 410-414 | 410-414 | I20-I25 |
| Cerebrovascular disease/Stroke | 430-438 | 430-438 | I60-I69 |
| **Cancer** | 140-239 | 140-239 | C00-C97, D00-D48 |
| Lung cancer | 162 | 162 | C33-C34 |
| Breast cancer | 174 | 174-175 | C50 |
| Prostate cancer | 185 | 185 | C61 |
| Pancreas cancer | 157 | 157 | C25 |
| Colorectum cancer | 153-154 | 153-154 | C18-C21 |
| Stomach cancer | 151 | 151 | C16 |
| **Injury and poisoning** | N800-N999 | 800-999 | V01-V99, W00-W99, X00-X99, Y00-Y98 |
| **Respiratory disease** | 460-519 | 460-519 | J00-J99 |
| Chronic obstructive pulmonary disease | 490-493, 518 | 490-494, 496 | J40-J47 |
| Influenza/pneumonia | 470-474, 480-483, 485-486 | 480-487 | J10-J18 |
| **Mental disorders and Alzheimer’s disease** | 290-315 | 290-319, 331 | F00-F99, G30, G31 |
| Dementia | 290 | 290 | F01, F03 |
| Alzheimer’s disease | 293 | 331 | G30, G31 |
| **Other** |  |  |  |
| Diabetes mellitus | 250 | 250 | E10-E14 |
| Infectious and parasitic diseases | 000-136 | 001-139 | A00-B99 |

| **Table S2.** Goodness-of-fit criteria for different latent class models of socioeconomic trajectories in men (n=5729) | | | | | |
| --- | --- | --- | --- | --- | --- |
|  | 2 class | 3 class | 4 class | 5 class | 6 class |
| Degrees of freedom | 63 | 54 | 45 | 36 | 27 |
| Log likelihood | -19348.51 | -18692.003 | -18395.457 | -18198.826 | -18162.437 |
| AIC | 2517.7423 | 1222.7274 | 647.63696 | 272.37392 | 217.59694 |
| BIC | 2630.8483 | 1395.7131 | 880.50232 | 565.11896 | 570.22165 |
| Adjusted BIC | 2576.8273 | 1313.0927 | 769.28266 | 425.29995 | 401.80329 |
| Entropy | 0.70536427 | 0.74650066 | 0.73271695 | 0.68607425 | 0.67773044 |
| Posterior probabilities of class membership | 0.74  0.26 | 0.58  0.39  0.03 | 0.33  0.26  0.40  0.02 | 0.33  0.15  0.29  0.13  0.09 | 0.20  0.09  0.09  0.22  0.27  0.13 |
| AIC, Akaike’s Information Criterion; BIC, Bayesian Information Criterion | | | | | |

| **Table S3.** Goodness-of-fit criteria for different latent class models of socioeconomic trajectories in women (n=5607) | | | | | |
| --- | --- | --- | --- | --- | --- |
|  | 2 class | 3 class | 4 class | 5 class | 6 class |
| Degrees of freedom | 63 | 54 | 45 | 36 | 27 |
| Log likelihood | -18963.211 | -18355.415 | -18123.352 | -18045.66 | -18021.404 |
| AIC | 1994.5212 | 796.92893 | 350.80345 | 213.41925 | 182.90612 |
| BIC | 2107.2613 | 969.35497 | 582.91544 | 505.21717 | 534.38999 |
| Adjusted BIC | 2053.2404 | 886.73485 | 471.69604 | 365.3985 | 365.97204 |
| Entropy | 0.94560433 | 0.75442516 | 0.71901988 | 0.64620555 | 0.65346592 |
| Posterior probabilities of class membership | 0.91  0.09 | 0.56  0.35  0.09 | 0.26  0.38  0.27  0.09 | 0.24  0.24  0.27  0.17  0.09 | 0.12  0.07  0.09  0.28  0.22  0.22 |
| AIC, Akaike’s Information Criterion; BIC, Bayesian Information Criterion | | | | | |

| **Table S4.** The distribution (%) of missing cases of socioeconomic position according to socioeconomic position at birth and in adulthood (based on the study sample who survived till the start of follow up in 1980) | | | | | | |
| --- | --- | --- | --- | --- | --- | --- |
| **Men** | **SEP at Age 10** | | **SEP at ages 51-65** | | **Life course SEP** | |
|  | Non-missing | Missing | Non-missing | Missing | Non-missing | Missing* |
| **SEP at birth** |  |  |  |  |  |  |
| Low | 77.19 | 22.81 | 90.57 | 9.43 | 67.96 | 32.04 |
| Middle | 82.86 | 17.14 | 93.61 | 6.39 | 75.64 | 24.36 |
| High | 66.87 | 33.13 | 93.82 | 6.18 | 61.24 | 38.76 |
| P-value | 0.001 | | 0.001 | | 0.001 | |
| **Adult SEP at ages 31-45** |  |  |  |  |  |  |
| Low | 78.45 | 21.55 | 88.05 | 11.95 | 69.69 | 30.31 |
| Middle | 80.84 | 19.16 | 91.88 | 8.12 | 74.67 | 25.33 |
| High | 79.71 | 20.29 | 90.96 | 9.04 | 72.80 | 27.20 |
| P-value | 0.269 | | 0.001 | | 0.006 | |
| **Women** |  |  |  |  |  |  |
| **SEP at birth** |  |  |  |  |  |  |
| Low | 75.74 | 24.26 | 90.57 | 9.43 | 63.36 | 36.64 |
| Middle | 80.10 | 19.90 | 93.61 | 6.39 | 69.19 | 30.82 |
| High | 69.23 | 30.77 | 93.82 | 6.18 | 58.97 | 41.03 |
| P-value | 0.001 | | 0.001 | | 0.001 | |
| **Adult SEP at ages 31-45** |  |  |  |  |  |  |
| Low | 75.93 | 24.07 | 87.43 | 12.57 | 64.85 | 35.15 |
| Middle | 77.76 | 22.24 | 86.51 | 13.49 | 66.02 | 33.92 |
| High | 78.59 | 21.41 | 89.33 | 10.67 | 69.42 | 30.58 |
| P-value | 0.127 | | 0.028 | | 0.005 | |
| *Indicates missing data on SEP in at least one of the four measures of SEP at birth, age 10, ages 31-45, and ages 51-65, respectively. | | | | | | |

| **Table S5.** The distribution of major diagnoses within the domains of cause-specific mortality | | | | | |
| --- | --- | --- | --- | --- | --- |
| **Type of diagnoses** | **Men (n=5729)** | | **Women (n=5607)** | |  |
|  | No. of deaths | % (95% CI) | No. of deaths | % (95% CI) |  |
| **Cardiovascular disease** |  |  |  |  |  |
| Ischaemic heart disease | 1048 | 18.3 (17.3, 19.3) | 537 | 9.6 (8.8, 10.4) |  |
| Cerebrovascular disease/Stroke | 307 | 5.4 (4.8, 6.0) | 278 | 5.0 (4.4, 5.6) |  |
| **Cancer** |  |  |  |  |  |
| Lung cancer | 202 | 3.5 (3.1, 4.0) | 122 | 2.2 (1.8, 2.6) |  |
| Breast cancer | 1 | 0.0 (0.0, 0.1) | 126 | 2.2 (1.9, 2.7) |  |
| Prostate cancer | 231 | 4.0 (3.6, 4.6) | 0 | 0 |  |
| Pancreas cancer | 73 | 1.3 (1.0, 1.6) | 88 | 1.6 (1.3, 1.9) |  |
| Colorectum cancer | 103 | 1.8 (1.5, 2.2) | 79 | 1.4 (1.1, 1.8) |  |
| Stomach cancer | 44 | 0.8 (0.6, 1.0) | 25 | 0.5 (0.3, 0.6) |  |
| **Injury and poisoning** | 136 | 2.4 (2.0, 2.8) | 86 | 1.5 (1.2, 1.9) |  |
| **Respiratory disease** |  |  |  |  |  |
| Chronic obstructive pulmonary disease | 111 | 1.9 (1.6, 2.3) | 121 | 2.2 (1.8, 2.6) |  |
| Influenza/pneumonia | 88 | 1.5 (1.2, 1.9) | 56 | 1.0 (0.8, 1.3) |  |
| **Mental disorders and Alzheimer’s disease** |  |  |  |  |  |
| Dementia | 65 | 1.1 (0.9, 1.4) | 93 | 1.7 (1.4, 2.0) |  |
| Alzheimer’s disease | 36 | 0.6 (0.5, 0.8) | 59 | 1.0 (0.8, 1.4) |  |
| **Other** |  |  |  |  |  |
| Diabetes mellitus | 85 | 2.2 (1.8, 2.8) | 57 | 1.9 (1.5, 2.5) |  |
| Infectious and parasitic diseases | 40 | 0.7 (0.5, 0.9) | 43 | 0.8 (0.6, 1.0) |  |

| **Table S6**. Hazard ratios (95% CI) of the associations between latent class trajectories of socioeconomic position and mortality in men aged 51-95 years: contribution of school grades (n=4716) | | | | | | | |
| --- | --- | --- | --- | --- | --- | --- | --- |
| **Latent class trajectories of socioeconomic position** | **Mortality** | | | | | | |
|  | **All-cause** | **Cardiovascular disease** | **Cancer** | **Injuries & poisoning** | **Respiratory disease** | **Mental disorders** | **Other** |
|  | HR (95% CI) | HR (95% CI) | HR (95% CI) | HR (95% CI) | HR (95% CI) | HR (95% CI) | HR (95% CI) |
| No. of death | 3178 | 1532 | 891 | 108 | 189 | 108 | 350 |
| **Model 1** |  |  |  |  |  |  |  |
| Stable low (Reference) | 1.00 | 1.00 | 1.00 | 1.00 | 1.00 | 1.00 | 1.00 |
| Upward from low | 0.81 (0.73, 0.90) | 0.84 (0.72, 0.98) | 0.89 (0.72, 1.09) | 0.59 (0.32, 1.07) | 0.71 (0.45, 1.13) | 0.74 (0.40, 1.37) | 0.67 (0.48, 0.94) |
| Stable middle/ middle to low | 0.85 (0.78, 0.92) | 0.79 (0.70, 0.90) | 0.97 (0.82, 1.15) | 0.70 (0.45, 1.11) | 0.99 (0.71, 1.39) | 0.97 (0.60, 1.55) | 0.80 (0.61, 1.03) |
| Upward from middle | 0.73 (0.65, 0.82) | 0.70 (0.59, 0.83) | 0.93 (0.76, 1.15) | 0.37 (0.17, 0.78) | 0.46 (0.26, 0.80) | 0.79 (0.42, 1.47) | 0.72 (0.52, 1.02) |
| Stable high | 0.78 (0.68, 0.90) | 0.68 (0.55, 0.84) | 0.97 (0.75, 1.25) | 0.74 (0.36, 1.52) | 0.60 (0.32, 1.13) | 1.03 (0.51, 2.08) | 0.85 (0.57, 1.27) |
| p-value for heterogeneity* | 0.001 | 0.001 | 0.850 | 0.069 | 0.023 | 0.828 | 0.114 |
|  |  |  |  |  |  |  |  |
| **Model 2** |  |  |  |  |  |  |  |
| Stable low (Reference) | 1.00 | 1.00 | 1.00 | 1.00 | 1.00 | 1.00 | 1.00 |
| Upward from low | 0.80 (0.70, 0.93) | 0.86 (0.73, 0.99) | 0.89 (0.72, 1.10) | 0.62 (0.34, 1.13) | 0.72 (0.46, 1.15) | 0.76 (0.41, 1.43) | 0.71 (0.50, 1.00) |
| Stable middle/ middle to low | 0.86 (0.79, 0.94) | 0.80 (0.71, 0.91) | 0.97 (0.82, 1.15) | 0.72 (0.46, 1.14) | 1.00 (0.72, 1.40) | 0.99 (0.62, 1.59) | 0.82 (0.63, 1.07) |
| Upward from middle | 0.76 (0.68, 0.85) | 0.72 (0.61, 0.86) | 0.94 (0.76, 1.16) | 0.39 (0.18, 0.85) | 0.46 (0.26, 0.82) | 0.84 (0.45, 1.58) | 0.80 (0.57, 1.13) |
| Stable high | 0.81 (0.70, 0.93) | 0.70 (0.56, 0.87) | 0.97 (0.75, 1.26) | 0.80 (0.39, 1.66) | 0.61 (0.32, 1.16) | 1.10 (0.54, 2.25) | 0.94 (0.62, 1.41) |
| p-value for heterogeneity* | 0.001 | 0.001 | 0.870 | 0.129 | 0.033 | 0.874 | 0.293 |
| HR=Hazard Ratio; CI= Confidence Interval  Model 1 minimally adjusted for age and birth cohort (1915-1919, 1920-1924, 1925-1929); Model 2 additionally adjusted for the standardized mean of the school grades.  *p-value obtained from Wald test to test the significance of the overall association.  Note: Mortality from mental disorders includes Alzheimer’s disease mortality | | | | | | | |

| **Table S7**. Hazard ratios (95% CI) of the associations between latent class trajectories of socioeconomic position and mortality in women aged 51-95 years: contribution of school grades (n=4642) | | | | | | | |
| --- | --- | --- | --- | --- | --- | --- | --- |
| **Latent class trajectories of socioeconomic position** | **Mortality** | | | | | | |
|  | **All-cause** | **Cardiovascular disease** | **Cancer** | **Injuries & poisoning** | **Respiratory disease** | **Mental disorders** | **Other** |
|  | HR (95% CI) | HR (95% CI) | HR (95% CI) | HR (95% CI) | HR (95% CI) | HR (95% CI) | HR (95% CI) |
| No. of death | 2469 | 1042 | 774 | 69 | 163 | 132 | 289 |
| **Model 1** |  |  |  |  |  |  |  |
| Stable low (Reference) | 1.00 | 1.00 | 1.00 | 1.00 | 1.00 | 1.00 | 1.00 |
| Upward from low | 0.77 (0.65, 0.90) | 0.76 (0.59, 1.99) | 0.84 (0.62, 1.12) | 0.27 (0.06, 1.13) | 1.04 (0.61, 1.75) | 0.59 (0.27, 1.26) | 0.64 (0.39, 1.06) |
| Stable middle/ middle to low | 0.67 (0.59, 0.76) | 0.67 (0.55, 0.81) | 0.77 (0.62, 0.96) | 0.79 (0.41, 1.52) | 0.57 (0.36, 0.90) | 0.59 (0.34, 1.00) | 0.54 (0.37, 0.78) |
| Upward from middle | 0.79 (0.71, 0.88) | 0.85 (0.73, 1.00) | 0.79 (0.65, 0.96) | 0.78 (0.43, 1.41) | 0.47 (0.31, 0.73) | 0.82 (0.53, 1.27) | 0.77 (0.57, 1.04) |
| Stable high | 0.70 (0.62, 0.78) | 0.72 (0.60, 0.86) | 0.83 (0.68, 1.01) | 0.32 (0.14, 0.73) | 0.49 (0.32, 0.77) | 0.55 (0.33, 0.91) | 0.63 (0.45, 0.88) |
| p-value for heterogeneity* | 0.001 | 0.001 | 0.096 | 0.046 | 0.001 | 0.111 | 0.008 |
|  |  |  |  |  |  |  |  |
| **Model 2** |  |  |  |  |  |  |  |
| Stable low (Reference) | 1.00 | 1.00 | 1.00 | 1.00 | 1.00 | 1.00 | 1.00 |
| Upward from low | 0.78 (0.66, 0.92) | 0.78 (0.60, 1.01) | 0.84 (0.62, 1.13) | 0.28 (0.06, 1.18) | 1.10 (0.65, 1.86) | 0.58 (0.27, 1.25) | 0.66 (0.40, 1.09) |
| Stable middle/ middle to low | 0.68 (0.60, 0.78) | 0.68 (0.56, 0.83) | 0.77 (0.62, 0.96) | 0.81 (0.42, 1.57) | 0.59 (0.37, 0.95) | 0.58 (0.34, 1.00) | 0.55 (0.37, 0.80) |
| Upward from middle | 0.80 (0.72, 0.89) | 0.87 (0.74, 1.02) | 0.79 (0.65, 0.96) | 0.80 (0.44, 1.44) | 0.49 (0.32, 0.76) | 0.81 (0.52, 1.26) | 0.78 (0.57, 1.05) |
| Stable high | 0.70 (0.63, 0.79) | 0.73 (0.61, 0.87) | 0.83 (0.68, 1.01) | 0.33 (0.15, 0.74) | 0.51 (0.32, 0.80) | 0.54 (0.33, 0.91) | 0.64 (0.46, 0.89) |
| p-value for heterogeneity* | 0.001 | 0.001 | 0.098 | 0.052 | 0.001 | 0.107 | 0.012 |
| HR=Hazard Ratio; CI= Confidence Interval  Model 1 minimally adjusted for age and birth cohort (1915-1919, 1920-1924, 1925-1929); Model 2 additionally adjusted for the standardized mean of the school grades.  *p-value obtained from Wald test to test the significance of the overall association.  Note: Mortality from mental disorders includes Alzheimer’s disease mortality | | | | | | | |
