# Supplementary material for: Latent class trajectories of socioeconomic position over four time points and mortality: the Uppsala Birth Cohort Study
Source: Eur J Public Health. 2022 Jul 5;32(4):522–7. doi: 10.1093/eurpub/ckac060 (PMC9341739; doi:10.1093/eurpub/ckac060)
Supplement: ckac060_Supplementary_Data [file ckac060_supplementary_data.zip › updated _Supplementary file.pdf]

Supplementary Contents

Additional References: 41 through 49

Figure S1. Latent class trajectories of socioeconomic position in men (n=5729)

Figure S2. Latent class trajectories of socioeconomic position in women (n=5607)

Table S1. International Classification of Disease Codes for broad groups of mortality with major diagnoses in each group

Table S2. Goodness-of-fit criteria for different latent class models of socioeconomic trajectories in men (n=5729)

Table S3. Goodness-of-fit criteria for different latent class models of socioeconomic trajectories in women (n=5607)

Table S4. The distribution (%) of missing cases of socioeconomic position according to socioeconomic position at birth and in adulthood (based on the study sample who survived till the start of follow up in 1980)

Table S5. The distribution of major diagnoses within the domains of cause-specific mortality

Table S6. Hazard ratios (95% CI) of the associations between latent class trajectories of socioeconomic position and mortality in men aged 51-95 years: contribution of school grades (n=4716)

Table S7. Hazard ratios (95% CI) of the associations between latent class trajectories of socioeconomic position and mortality in women aged 51-95 years: contribution of school grades (n=4642

Additional References: 41 through 49

41. Gall SL, Abbott-Chapman J, Patton GC, Dwyer T, Venn A. Intergenerational educational mobility is associated with cardiovascular disease risk behaviours in a cohort of young Australian adults: The Childhood Determinants of Adult Health (CDAH) Study. BMC Public Health. 2010;10.

42. Sorokin PA. Social and Cultural Mobility. 4th ed. New York: Free Press; 1959. Pp. 99–145

43. Jonsson F, Sebastian MS, Hammarström A, Gustafsson PE. Intragenerational social mobility and functional somatic symptoms in a northern Swedish context: analyses of diagonal reference models. Int J Equity Health. 2017;16(1):1–10.

44. Chan TW. Social mobility and the well-being of individuals. Br J Sociol. 2018;69(1):183–206.

45. Ellis RA, Lane WC. Social mobility and social isolation: A test of Sorokin's dissociative hypothesis. Am Sociol Rev. 1967;32(2):237.

46. Brooke HL, Talbäck M, Hörnblad J, Johansson LA, Ludvigsson JF, Druid H, et al. The Swedish cause of death register. Eur J Epidemiol. 2017;32(9):765–73.

47. Davies CE, Glonek GFV, Giles LC. The impact of covariance misspecification in group-based trajectory models for longitudinal data with non-stationary covariance structure. Stat Methods Med Res. 2017;26(4):1982–91.

48. Enders CK, Bandalos DL. The relative performance of full information maximum likelihood estimation for missing data in structural equation models. Struct Equ Model. 2001;8(3):430–57.

49. Billingsley S. Sick leave absence and the relationship between intra-generational social mobility and mortality: Health selection in Sweden. BMC Public Health. 2020;20(1):1–15.

Supplementary Figures

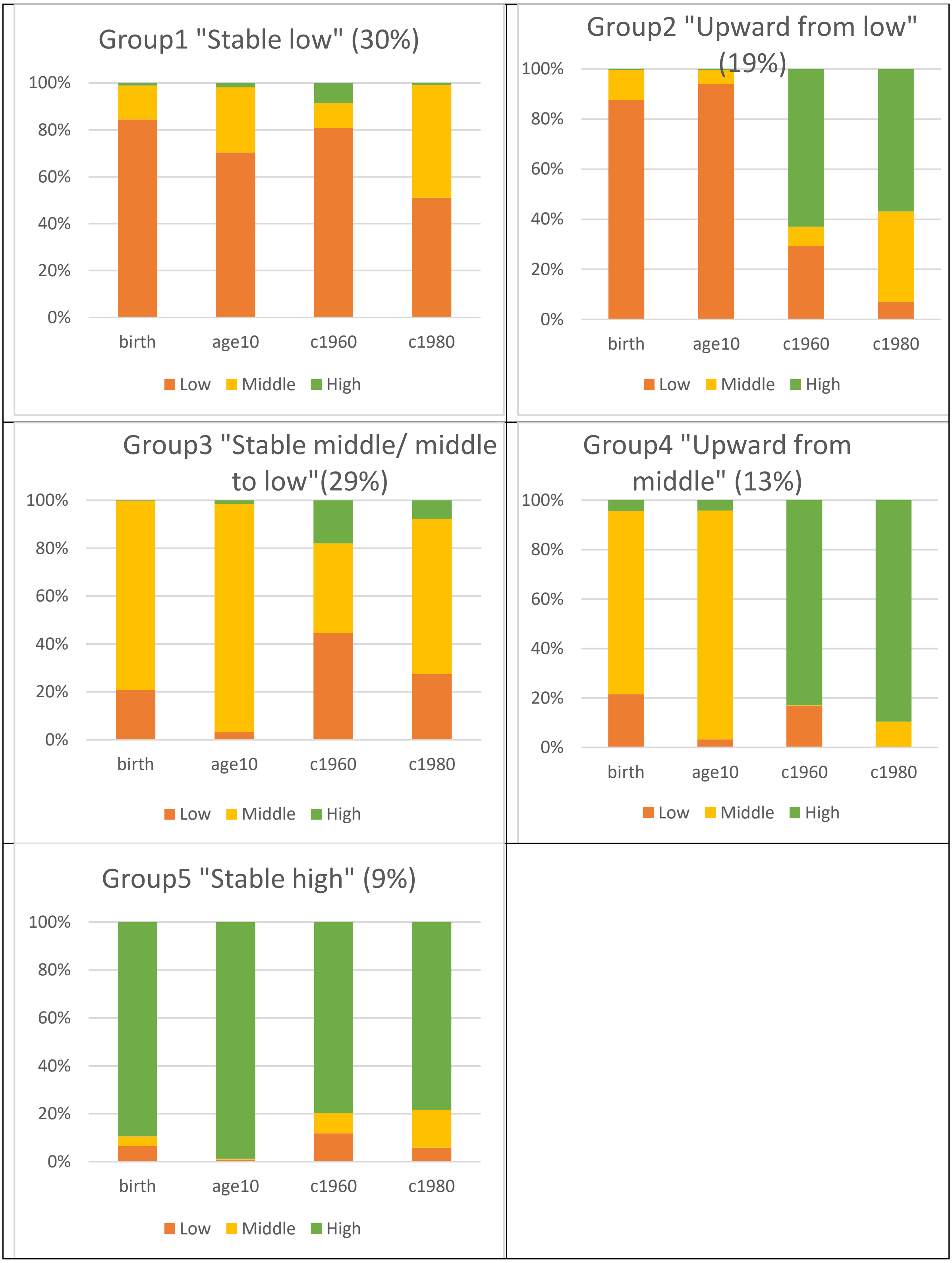

Figure S1. Latent class trajectories of socioeconomic position in men (n=5729)

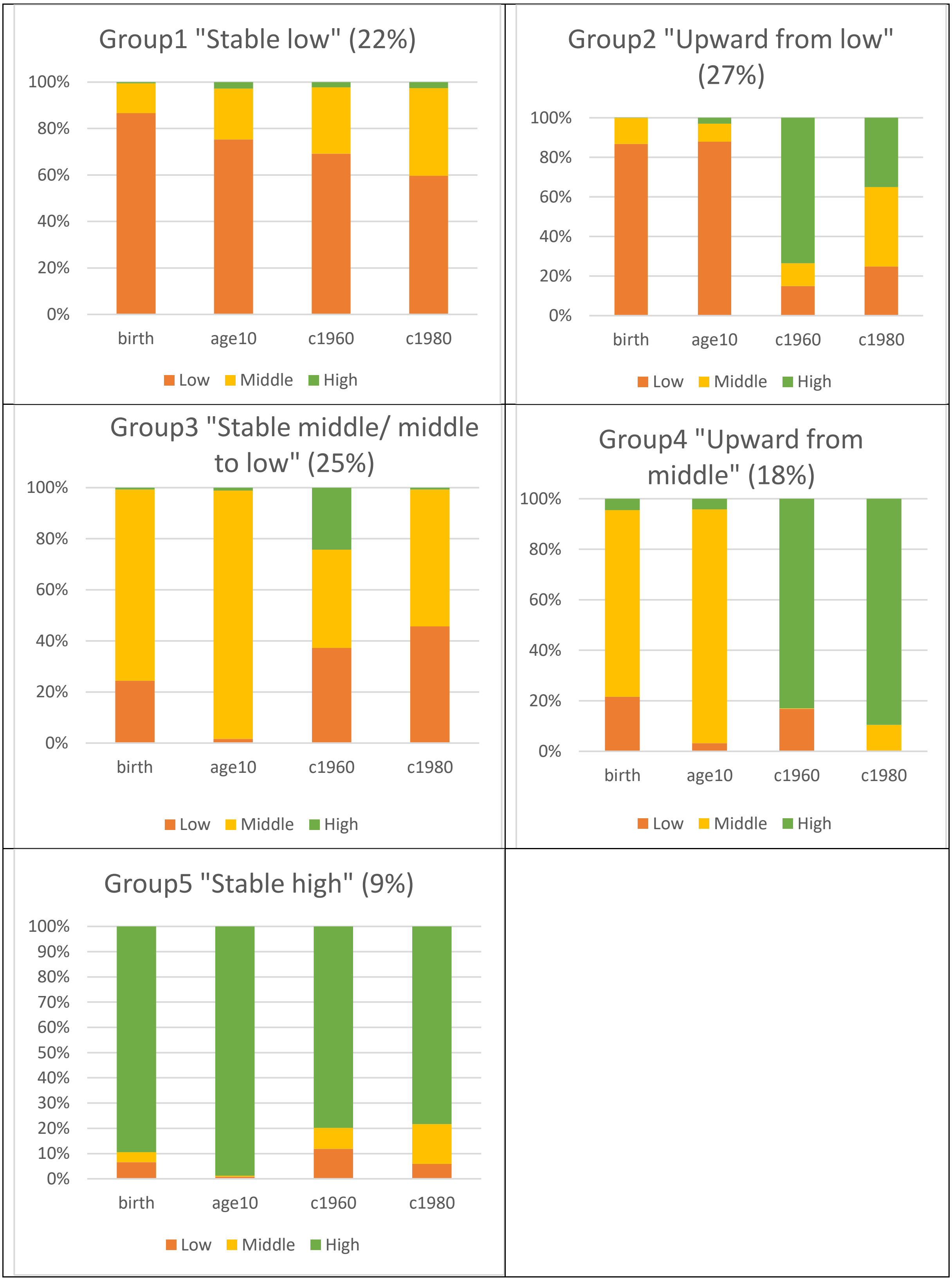

Figure S2. Latent class trajectories of socioeconomic position in women (n=5607)

Supplementary Tables

Table S1. International Classification of Disease Codes for broad groups of mortality with major diagnoses in each group

	**ICD-8** (1980-1986)	**ICD-9** (1987-1996)	**ICD-10** (1997-2009)
**Cardiovascular disease**	390-458	390-459	I00-I99
Ischaemic heart disease	410-414	410-414	I20-I25
Cerebrovascular disease/Stroke	430-438	430-438	I60-I69
**Cancer**	140-239	140-239	C00-C97, D00-D48
Lung cancer	162	162	C33-C34
Breast cancer	174	174-175	C50
Prostate cancer	185	185	C61
Pancreas cancer	157	157	C25
Colorectum cancer	153-154	153-154	C18-C21
Stomach cancer	151	151	C16
**Injury and poisoning**	N800-N999	800-999	V01-V99, W00-W99, X00-X99, Y00-Y98
**Respiratory disease**	460-519	460-519	J00-J99
Chronic obstructive pulmonary disease	490-493, 518	490-494, 496	J40-J47
Influenza/pneumonia	470-474, 480-483, 485-486	480-487	J10-J18
**Mental disorders and Alzheimer's disease**	290-315	290-319, 331	F00-F99, G30, G31
Dementia	290	290	F01, F03
Alzheimer's disease	293	331	G30, G31
**Other**			
Diabetes mellitus	250	250	E10-E14
Infectious and parasitic diseases	000-136	001-139	A00-B99

Table S2. Goodness-of-fit criteria for different latent class models of socioeconomic trajectories in men (n=5729)

	2 class	3 class	4 class	5 class	6 class
Degrees of freedom	63	54	45	36	27
Log likelihood	-19348.51	-18692.003	-18395.457	-18198.826	-18162.437
AIC	2517.7423	1222.7274	647.63696	272.37392	217.59694
BIC	2630.8483	1395.7131	880.50232	565.11896	570.22165
Adjusted BIC	2576.8273	1313.0927	769.28266	425.29995	401.80329
Entropy	0.70536427	0.74650066	0.73271695	0.68607425	0.67773044
Posterior probabilities of class membership	0.74	0.58	0.33	0.33	0.20
	0.26	0.39	0.26	0.15	0.09
		0.03	0.40	0.29	0.09
			0.02	0.13	0.22
				0.09	0.27
					0.13

AIC, Akaike's Information Criterion; BIC, Bayesian Information Criterion

**Table S3.** Goodness-of-fit criteria for different latent class models of socioeconomic trajectories in women (n=5607)					
	2 class	3 class	4 class	5 class	6 class
Degrees of freedom	63	54	45	36	27
Log likelihood	-18963.211	-18355.415	-18123.352	-18045.66	-18021.404
AIC	1994.5212	796.92893	350.80345	213.41925	182.90612
BIC	2107.2613	969.35497	582.91544	505.21717	534.38999
Adjusted BIC	2053.2404	886.73485	471.69604	365.3985	365.97204
Entropy	0.94560433	0.75442516	0.71901988	0.64620555	0.65346592
Posterior probabilities of class	0.91	0.56	0.26	0.24	0.12
membership	0.09	0.35	0.38	0.24	0.07
		0.09	0.27	0.27	0.09
			0.09	0.17	0.28
				0.09	0.22
					0.22

AIC, Akaike's Information Criterion; BIC, Bayesian Information Criterion

Table S4. The distribution (%) of missing cases of socioeconomic position according to socioeconomic position at birth and in adulthood (based on the study sample who survived till the start of follow up in 1980)

	**SEP at Age 10**		**SEP at ages 51-65**		**Life course SEP**	
**Men**	Non-missing	Missing	Non-missing	Missing	Non-missing	Missing*
**SEP at birth**						
Low	77.19	22.81	90.57	9.43	67.96	32.04
Middle	82.86	17.14	93.61	6.39	75.64	24.36
High	66.87	33.13	93.82	6.18	61.24	38.76
P-value	0.001		0.001		0.001	
**Adult SEP at ages 31-45**						
Low	78.45	21.55	88.05	11.95	69.69	30.31
Middle	80.84	19.16	91.88	8.12	74.67	25.33
High	79.71	20.29	90.96	9.04	72.80	27.20
P-value	0.269		0.001		0.006	
**Women**						
**SEP at birth**						
Low	75.74	24.26	90.57	9.43	63.36	36.64
Middle	80.10	19.90	93.61	6.39	69.19	30.82
High	69.23	30.77	93.82	6.18	58.97	41.03
P-value	0.001		0.001		0.001	
**Adult SEP at ages 31-45**						
Low	75.93	24.07	87.43	12.57	64.85	35.15
Middle	77.76	22.24	86.51	13.49	66.02	33.92
High	78.59	21.41	89.33	10.67	69.42	30.58
P-value	0.127		0.028		0.005	

*Indicates missing data on SEP in at least one of the four measures of SEP at birth, age 10, ages 31-45, and ages 51-65, respectively.

Table S5. The distribution of major diagnoses within the domains of cause-specific mortality

Type of diagnoses	Men (n=5729)		Women (n=5607)	
	No. of deaths	% (95% CI)	No. of deaths	% (95% CI)
**Cardiovascular disease**				
Ischaemic heart disease	1048	18.3 (17.3, 19.3)	537	9.6 (8.8, 10.4)
Cerebrovascular disease/Stroke	307	5.4 (4.8, 6.0)	278	5.0 (4.4, 5.6)
**Cancer**				
Lung cancer	202	3.5 (3.1, 4.0)	122	2.2 (1.8, 2.6)
Breast cancer	1	0.0 (0.0, 0.1)	126	2.2 (1.9, 2.7)
Prostate cancer	231	4.0 (3.6, 4.6)	0	0
Pancreas cancer	73	1.3 (1.0, 1.6)	88	1.6 (1.3, 1.9)
Colorectum cancer	103	1.8 (1.5, 2.2)	79	1.4 (1.1, 1.8)
Stomach cancer	44	0.8 (0.6, 1.0)	25	0.5 (0.3, 0.6)
**Injury and poisoning**	136	2.4 (2.0, 2.8)	86	1.5 (1.2, 1.9)
**Respiratory disease**				
Chronic obstructive pulmonary disease	111	1.9 (1.6, 2.3)	121	2.2 (1.8, 2.6)
Influenza/pneumonia	88	1.5 (1.2, 1.9)	56	1.0 (0.8, 1.3)
**Mental disorders and Alzheimer's disease**				
Dementia	65	1.1 (0.9, 1.4)	93	1.7 (1.4, 2.0)
Alzheimer's disease	36	0.6 (0.5, 0.8)	59	1.0 (0.8, 1.4)
**Other**				
Diabetes mellitus	85	2.2 (1.8, 2.8)	57	1.9 (1.5, 2.5)
Infectious and parasitic diseases	40	0.7 (0.5, 0.9)	43	0.8 (0.6, 1.0)

Table S6. Hazard ratios (95% CI) of the associations between latent class trajectories of socioeconomic position and mortality in men aged 51-95 years: contribution of school grades (n=4716)

	Mortality						
**Latent class trajectories of socioeconomic position**	**All-cause**	**Cardiovascular disease**	**Cancer**	**Injuries & poisoning**	**Respiratory disease**	**Mental disorders**	**Other**
	HR (95% CI)	HR (95% CI)	HR (95% CI)	HR (95% CI)	HR (95% CI)	HR (95% CI)	HR (95% CI)
No. of death	3178	1532	891	108	189	108	350
**Model 1**							
Stable low (Reference)	1.00	1.00	1.00	1.00	1.00	1.00	1.00
Upward from low	0.81 (0.73, 0.90)	0.84 (0.72, 0.98)	0.89 (0.72, 1.09)	0.59 (0.32, 1.07)	0.71 (0.45, 1.13)	0.74 (0.40, 1.37)	0.67 (0.48, 0.94)
Stable middle/ middle to low	0.85 (0.78, 0.92)	0.79 (0.70, 0.90)	0.97 (0.82, 1.15)	0.70 (0.45, 1.11)	0.99 (0.71, 1.39)	0.97 (0.60, 1.55)	0.80 (0.61, 1.03)
Upward from middle	0.73 (0.65, 0.82)	0.70 (0.59, 0.83)	0.93 (0.76, 1.15)	0.37 (0.17, 0.78)	0.46 (0.26, 0.80)	0.79 (0.42, 1.47)	0.72 (0.52, 1.02)
Stable high	0.78 (0.68, 0.90)	0.68 (0.55, 0.84)	0.97 (0.75, 1.25)	0.74 (0.36, 1.52)	0.60 (0.32, 1.13)	1.03 (0.51, 2.08)	0.85 (0.57, 1.27)
p-value for heterogeneity*	0.001	0.001	0.850	0.069	0.023	0.828	0.114
**Model 2**							
Stable low (Reference)	1.00	1.00	1.00	1.00	1.00	1.00	1.00
Upward from low	0.80 (0.70, 0.93)	0.86 (0.73, 0.99)	0.89 (0.72, 1.10)	0.62 (0.34, 1.13)	0.72 (0.46, 1.15)	0.76 (0.41, 1.43)	0.71 (0.50, 1.00)
Stable middle/ middle to low	0.86 (0.79, 0.94)	0.80 (0.71, 0.91)	0.97 (0.82, 1.15)	0.72 (0.46, 1.14)	1.00 (0.72, 1.40)	0.99 (0.62, 1.59)	0.82 (0.63, 1.07)
Upward from middle	0.76 (0.68, 0.85)	0.72 (0.61, 0.86)	0.94 (0.76, 1.16)	0.39 (0.18, 0.85)	0.46 (0.26, 0.82)	0.84 (0.45, 1.58)	0.80 (0.57, 1.13)
Stable high	0.81 (0.70, 0.93)	0.70 (0.56, 0.87)	0.97 (0.75, 1.26)	0.80 (0.39, 1.66)	0.61 (0.32, 1.16)	1.10 (0.54, 2.25)	0.94 (0.62, 1.41)
p-value for heterogeneity*	0.001	0.001	0.870	0.129	0.033	0.874	0.293

HR=Hazard Ratio; CI= Confidence Interval

Model 1 minimally adjusted for age and birth cohort (1915-1919, 1920-1924, 1925-1929); Model 2 additionally adjusted for the standardized mean of the school grades.

*p-value obtained from Wald test to test the significance of the overall association.

Note: Mortality from mental disorders includes Alzheimer's disease mortality

Table S7. Hazard ratios (95% CI) of the associations between latent class trajectories of socioeconomic position and mortality in women aged 51-95 years: contribution of school grades (n=4642)

**Latent class trajectories of socioeconomic position**	**Mortality**						
	**All-cause**	**Cardiovascular disease**	**Cancer**	**Injuries & poisoning**	**Respiratory disease**	**Mental disorders**	**Other**
	HR (95% CI)	HR (95% CI)	HR (95% CI)	HR (95% CI)	HR (95% CI)	HR (95% CI)	HR (95% CI)
No. of death	2469	1042	774	69	163	132	289
**Model 1**							
Stable low (Reference)	1.00	1.00	1.00	1.00	1.00	1.00	1.00
Upward from low	0.77 (0.65, 0.90)	0.76 (0.59, 1.99)	0.84 (0.62, 1.12)	0.27 (0.06, 1.13)	1.04 (0.61, 1.75)	0.59 (0.27, 1.26)	0.64 (0.39, 1.06)
Stable middle/ middle to low	0.67 (0.59, 0.76)	0.67 (0.55, 0.81)	0.77 (0.62, 0.96)	0.79 (0.41, 1.52)	0.57 (0.36, 0.90)	0.59 (0.34, 1.00)	0.54 (0.37, 0.78)
Upward from middle	0.79 (0.71, 0.88)	0.85 (0.73, 1.00)	0.79 (0.65, 0.96)	0.78 (0.43, 1.41)	0.47 (0.31, 0.73)	0.82 (0.53, 1.27)	0.77 (0.57, 1.04)
Stable high	0.70 (0.62, 0.78)	0.72 (0.60, 0.86)	0.83 (0.68, 1.01)	0.32 (0.14, 0.73)	0.49 (0.32, 0.77)	0.55 (0.33, 0.91)	0.63 (0.45, 0.88)
p-value for heterogeneity*	0.001	0.001	0.096	0.046	0.001	0.111	0.008
**Model 2**							
Stable low (Reference)	1.00	1.00	1.00	1.00	1.00	1.00	1.00
Upward from low	0.78 (0.66, 0.92)	0.78 (0.60, 1.01)	0.84 (0.62, 1.13)	0.28 (0.06, 1.18)	1.10 (0.65, 1.86)	0.58 (0.27, 1.25)	0.66 (0.40, 1.09)
Stable middle/ middle to low	0.68 (0.60, 0.78)	0.68 (0.56, 0.83)	0.77 (0.62, 0.96)	0.81 (0.42, 1.57)	0.59 (0.37, 0.95)	0.58 (0.34, 1.00)	0.55 (0.37, 0.80)
Upward from middle	0.80 (0.72, 0.89)	0.87 (0.74, 1.02)	0.79 (0.65, 0.96)	0.80 (0.44, 1.44)	0.49 (0.32, 0.76)	0.81 (0.52, 1.26)	0.78 (0.57, 1.05)
Stable high	0.70 (0.63, 0.79)	0.73 (0.61, 0.87)	0.83 (0.68, 1.01)	0.33 (0.15, 0.74)	0.51 (0.32, 0.80)	0.54 (0.33, 0.91)	0.64 (0.46, 0.89)
p-value for heterogeneity*	0.001	0.001	0.098	0.052	0.001	0.107	0.012

HR=Hazard Ratio; CI= Confidence Interval

Model 1 minimally adjusted for age and birth cohort (1915-1919, 1920-1924, 1925-1929); Model 2 additionally adjusted for the standardized mean of the school grades.

*p-value obtained from Wald test to test the significance of the overall association.

Note: Mortality from mental disorders includes Alzheimer's disease mortality
